# A feed-forward regulation of endothelin receptors by c-Jun in human non-pigmented ciliary epithelial cells and retinal ganglion cells

**DOI:** 10.1371/journal.pone.0185390

**Published:** 2017-09-22

**Authors:** Junming Wang, Hai-Ying Ma, Raghu R. Krishnamoorthy, Thomas Yorio, Shaoqing He

**Affiliations:** 1 Department of Ophthalmology, Tongji Hospital, Tongji Medical College, Huazhong University of Science and Technology, Wuhan, Hubei, People's Republic of China; 2 North Texas Eye Research Institute, University of North Texas Health Science Center at Fort Worth, Fort Worth, Texas, United States of America; National Eye Centre, UNITED STATES

## Abstract

c-Jun, c-Jun N-terminal kinase(JNK) and endothelin B (ET_B_) receptor have been shown to contribute to the pathogenesis of glaucoma. Previously, we reported that an increase of c-Jun and CCAAT/enhancer binding protein β (C/EBPβ) immunohistostaining is associated with upregulation of the ET_B_ receptor within the ganglion cell layer of rats with elevated intraocular pressure (IOP). In addition, both transcription factors regulate the expression of the ET_B_ receptor in human non-pigmented ciliary epithelial cells (HNPE). The current study addressed the mechanisms by which ET-1 produced upregulation of ET receptors in primary rat retinal ganglion cells (RGCs) and HNPE cells. Treatment of ET-1 and ET-3 increased the immunocytochemical staining of c-Jun and C/EBPβ in primary rat RGCs and co-localization of both transcription factors was observed. A marked increase in DNA binding activity of AP-1 and C/EBPβ as well as elevated protein levels of c-Jun and c-Jun-N-terminal kinase (JNK) were detected following ET-1 treatment in HNPE cells. Overexpression of ET_A_ or ET_B_ receptor promoted the upregulation of c-Jun and also elevated its promoter activity. In addition, upregulation of C/EBPβ augmented DNA binding and mRNA expression of c-Jun, and furthermore, the interaction of c-Jun and C/EBPβ was confirmed using co-immunoprecipitation. Apoptosis of HNPE cells was identified following ET-1 treatment, and overexpression of the ET_A_ or ET_B_ receptor produced enhanced apoptosis. ET-1 mediated upregulation of c-Jun and C/EBPβ and their interaction may represent a novel mechanism contributing to the regulation of endothelin receptor expression. Reciprocally, c-Jun was also found to regulate the ET receptors and C/EBPβ appeared to play a regulatory role in promoting expression of c-Jun. Taken together, the data suggests that ET-1 triggers the upregulation of c-Jun through both ET_A_ and ET_B_ receptors, and conversely c-Jun also upregulates endothelin receptor expression, thereby generating a positive feed-forward loop of endothelin receptor activation and expression. This feed-forward regulation may contribute to RGC death and astrocyte proliferation following ET-1 treatment.

## Introduction

Glaucoma is a chronic eye disease affecting 70 million people [1] globally and is expected to reach 118 million by 2040 [2]. In recent decades, endothelins (ETs), a family of vasoactive peptides, and their receptors have been implicated as a key contributor to the etiology of glaucoma. ET-1 concentrations have been shown to be elevated in the aqueous humor and circulation of glaucoma patients, and in animal models of glaucoma (mouse, rat, dog, and monkey) [3–7]. Previously, we have demonstrated that elevation of ET-1 concentrations at the optic nerve head was associated with increased immunostaining of glial fibrillary acidic protein (GFAP, an astrocyte marker) in a rat model with high intraocular pressure [6]. In subsequent studies we found that ET-1 induced apoptosis of retinal ganglion cells in cultured primary rat retinal ganglion cells (RGCs) [8] and also in rats following intravitreal injection [9]. An elevation of ET_B_ receptor expression was also found in the Morrison’s ocular hypertension rat model [8], and the increased ET_B_ expression was associated with upregulation of transcription factors, AP-1 and C/EBPβ [10]. Both transcription factors have been shown to have regulatory roles in the cell cycle, growth, differentiation, proliferation and apoptosis [10–24]. c-Jun and C/EBPβ were found to be upregulated in response to elevated intraocular pressure (IOP) and co-localized at the ganglion cell layer in the rat retina [10]. It has been shown that c-Jun and its upstream kinase, JNK, are tightly associated with glaucomatous damage in several animal models [9, 25–27]. The binding sites of AP-1 and C/EBPβ are found in the promoter region of the ET_B_ receptor gene, and overexpression of either of them increases mRNA expression of the ET_A_ and ET_B_ receptors [10]. ETs bind to the ET receptor to trigger a variety of signaling pathways via G proteins, including Gαi, Gαs, Gαq, and Gβγ leading to the activation of downstream signaling pathways, including mitogen activated protein kinases (MAPKs), protein kinase C (PKC), and phosphatidylinositol-4,5-bisphosphate 3-kinase (PI3K) pathways. The different signaling pathways are also connected to a host of downstream transcriptional factors to exert ET’s function on gene expression. For instance, the phosphorylation of ERK1/2 is a key step in triggering downstream signaling and potential activation of transcriptional factors, such as c-Myc, Elk-1, c-Fos, AP-1, etc. [28–34].

Based on these initial findings we hypothesize that there is a feed-forward loop in the regulation of ET receptors and involve transcription factors, c-Jun/AP-1 and C/EBPβ. Furthermore, AP-1 and C/EBPβ have been found to be associated with elevated IOP in the Morrison’s rat model [10], and also co-localized in the retinal ganglion cell layer of rats [10]. An additional question addressed in the current study was whether AP-1 and C/EBPβ interact with one another to elicit their regulatory roles in gene expression. Herein, we used HNPE cells and primary RGCs to dissect the crosstalk between AP-1 and C/EBPβ, and to study the regulation between both transcription factors and ET receptors.

## Materials and methods

### Primary retinal ganglion cells (RGCs) isolation

RGCs from retinas of rat pups post natal 4–7 days were purified by a Thy-1.1 antibody-panning method [35] as described by Barres et al. [36]. Timed-pregnant Sprague-Dawley rats were purchased from Charles River Laboratories (Wilmington, MA, USA). All procedures were carried out in accordance with the ARVO Statement for the Use of Animals in Ophthalmic and Vision Research and approved by the Institutional Animal Care and Use Committee (IACUC) at University of North Texas Health Science Center at Fort Worth, TX, USA. Briefly, retinas were separated from the enucleated eyeballs of postnatal day 4 to 7 rat pups and dissociated using papain treatment. The cell suspension was panned with a rabbit anti-macrophage antibody (Cedarlane, Burlington, Ontario, Canada) to exclude macrophages followed by a panning with an anti-Thy1.1 antibody to selectively bind RGCs. The collected RGCs were seeded on glass coverslips coated with mouse-laminin and poly-D-lysine in serum-free Dulbecco’s modified Eagle’s medium containing brain-derived neurotrophic factor (50 ng/mL; Peprotech, Rocky Hill, NJ, USA), ciliary neurotrophic factor (10 ng/mL; Peprotech), and forskolin (5 ng/mL; Sigma-Aldrich Corp.). Cells were incubated at 37°C in a humidified atmosphere of 10% CO_2_ and 90% air. One-half volume of the culture medium was changed every two days.

### Cells culture and materials

A human non-pigmented ciliary epithelial (HNPE) cell line was a kind gift from Dr. Miguel Coca-Prados [37]. The cells were maintained using Dulbecco’s modified Eagle’s medium (DMEM) containing 10% fetal bovine serum in the presence of penicillin (100 μg/ml) and streptomycin (100 units/ml).

Primary antibodies were purchased from Santa Cruz Biotechnologies Inc. (c-Jun, #sc-1694; p-c-Jun, #sc-822; C/EBPβ, #sc-7962) and the secondary antibodies from Life Science (donkey anti-mouse Alexa 488, donkey anti-mouse Alexa 546 and donkey anti-rabbit 647-conjugate).

### Luciferase reporter assay

The 1043bp upstream promoter fragment of the human c-Jun promoter were generated by PCR amplification from human genomic DNA, and inserted into pGL3-Basic vector carrying the firefly luciferase reporter gene (Promega, Madison, WI) and named as pGL3-c-Jun. The pGL3 basic with SV40 promoter served as a positive control and pGL3-Basic without a promoter sequence as a negative control. The c-Jun promoter construct was confirmed by DNA sequencing. The human c-Jun, C/EBPβ, ET_A_ receptor, or ET_B_ receptor constructs were co-transfected with pGL3-Basic carrying c-Jun promoter for 24 hours. Luciferase activity was measured using Luciferase Assay System (Promega, Madison, WI). In brief, the collected cells were disrupted using a lysis buffer. The resultant cell lysate was mixed with luciferase assay reagent followed by brief vortexing, and luminescence value was measured immediately in Luminometer (Turner Biosystems). Assays were carried out in triplicate and mean values were normalized with protein amount of the corresponding samples. The relative fold increase in reporter activity was obtained by calculating the ratio of the activity of the promoter construct to that of the empty vector control.

### Electrophoretic mobility shift assay (EMSA) and supershift assay

Biotin-oligonucleotide labeling, binding, and electrophoresis were carried out according to the instruction of the manufacturer (Biotin 3´ End DNA Labeling Kit and LightShift Chemiluminescent EMSA Kit; Thermo Fisher Scientific, Inc., Waltham, MA); Oligonucleotides was synthesized from Integrated DNA Technologies, Inc. (Coralville, Iowa; AP-1: 5’-CGC TTG ATG ACT CAG CCG GAA-3’; C/EBP: 5’-TGC AGA TTG CGC AAT CTG CA-3’). The unlabeled oligonucleotides were used as specific competitors to determine the specificity of the binding. Nuclear extracts were prepared from HNPE cells. Nuclear extracts (10 μg) were incubated with 20 fmol of the Biotin-labelled probe in a reaction system (1×binding buffer, 2.5% glycerol, 5 mM MgCl_2_, 50ng/μL poly dI·dC) for 20 min at room temperature. The DNA-protein complex was separated on 6% native polyacrylamide gel. For competition or supershift assays, competitor (unlabeled probe) or 0.5 μg antibodies (antibody against c-Jun (from rabbit) or antibody against p-c-Jun (from mouse), Santa Cruz, CA) were pre-incubated with nuclear extracts for 10 min prior to addition of the probe.

### Co-Immunoprecipitation

Cell lysates were prepared in hypotonic buffer (20mM Tris pH 7.4, 10 mM EDTA, and cOmplete protease inhibitor cocktail (Roche Diagnostics, Indianapolis, IN)) by brief sonication of the collected cells. Each cell lysate (500μg of total protein) was precleared with 15 μl of Pierce Protein A/G Magnetic Beads (Thermo Fisher Scientific, Inc., Waltham, MA) for 2 hrs. The supernatants were collected and incubated with 1 μg of mouse monoclonal C/EBPβ by rotating at 4°C overnight, and the samples incubated with 1 μg anti-rabbit-IgG and anti-mouse-IgG were used as negative controls (Jackson ImmunoResearch Inc. West Grove, PA). Pre-washed magnetic Protein A/G beads (20 μl) were added into each of mixtures and incubated at room temperature for 2 hours with rotation. The beads were then collected with a magnetic stand and washed with 1 ml of washing buffer for five times. SDS-PAGE reducing sample buffer (100μl) was added to the tube, and the samples were incubated at room temperature for 10 minutes. The resultant supernatant containing target proteins and antibodies was subjected to 10% SDS-PAGE, and target proteins were analyzed by western blotting using an anti-c-Jun antibody.

### Quantitative real-time PCR

Total RNA was extracted using Trizol (Invitrogen Inc.) according to the manufacturer’s protocol and RNA concentration was determined using NanoDrop 2000 (Thermo Scientific Waltham, MA). cDNA was prepared from equal amount of total RNA using iScript Reverse Transcription Kit (Bio-Rad, Inc., Hercules, CA). SYBR Green-based real-time PCR was performed to detect gene expression using synthesized cDNA as template. Primers used in this study are listed below:

**Human c-Jun** (NM_002228.3):
  Forward: 5'- GACCTTCTATGACGATGCCC Reverse: 5'-AGGGTCATGCTCTGTTTCAG
**Human cyclophilin A** (NM_021130.3):
  Forward: 5’-TTCATCTGCACTGCCAAGAC Reverse: 5’-TGGAGTTGTCCACAGTCAGC

The real-time PCR was run in triplicate for each sample, and the assay was repeated 2 to 4 times. Cyclophilin A served as an internal control to normalize for equal loading of cDNA template. The results are presented as relative fold change compared to untreated control. The statistical significance was calculated by one-way ANOVA, and p values <0.05 were considered significant.

### Cells death analysis

The HNPE cells were seeded at equal number per well and cultured on glass coverslips at 60–80% confluence. Annexin V-FITC detection kit (Biotool Inc., Houston, TX) was used to detect apoptosis of cells. After treatments, the cells were washed twice with phosphate-buffered saline (PBS) followed by incubation with 100 μl binding buffer containing annexin V-FITC and propidium iodide (PI), at room temperature for 15 min in the dark. The fluorescence images were captured using a fluorescence microscope (Cytation 5, BioTek Inc.) with a dual filter set for FITC and rhodamine. The bright field (BF) images were taken for counting total cell number. BF images were processed by conversion to 8-bit grayscale, invert, and a slight adjustment of contrast. Nuclei were shown as a blob-like structure for manual counting using NIH ImageJ [38, 39].

### Immunofluorescent staining

Cells cultured on glass coverslips were treated following experimental designs and fixed in 4% paraformaldehyde in PBS for 15 minutes at room temperature. After permeabilization with 0.1% Triton X-100 for 15 minutes and blocking with 5% bovine serum albumin and 5% normal goat serum in PBS for 30 minutes, cells were incubated with the combination of primary antibodies at 4°C for overnight. Coverslips were then rinsed with PBS and incubated with corresponding secondary antibodies conjugated with either Alexa 488 or 647 for 1 hour at room temperature. Coverslips were mounted with ProLong Gold Antifade Mountant with DAPI (Invitrogen, Inc.) and subjected to confocal microscopy (LSM 510meta; Carl Zeiss, Dublin, CA). Images were captured with the same microscope settings.

## Results

### Co-localization of c-Jun and C/EBPβ in RGCs

**Fig 1:**
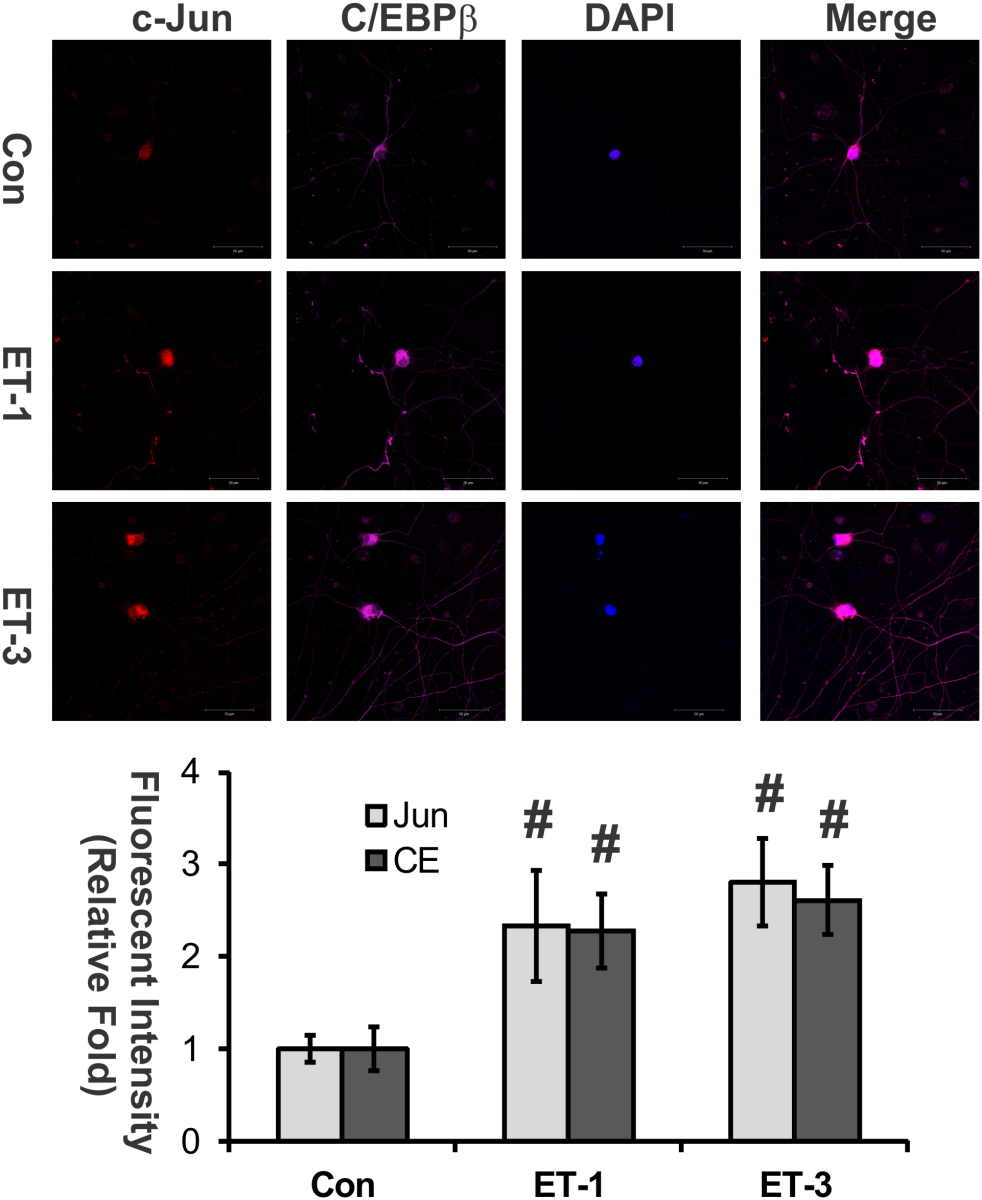
Co-localization of c-Jun and C/EBPβ in RGCs. Primary rat RGCs were cultured on glass coverslips for 7 days followed ET-1 or ET-3 treatment (100nM) for 24 hours. c-Jun and C/EBPβ (CE) protein levels were detected using immunocytochemistry in primary rat RGCs. Images were taken by using Zeiss Meta 510 con-focal microscope, and fluorescent intensity of staining was measured using NIH ImageJ. Flourescent staining of both proteins were overlaid in the merged images. The immunostaining of both factors was increased with treatment of ET-1 or ET-3. Bars represent mean and standard deviation (#: p< 0.01, one-way ANOVA, n = 6 from 2 individual experiments).

Since upregulation and co-localization of c-Jun and C/EBPβ were observed in the retinal ganglion cell layers of rats with elevated intraocular pressure (IOP) [10], we tested c-Jun and C/EBPβ protein levels by immunocytochemistry in primary rat RGCs treated with ET-1 or ET-3 for 24 hours. Both proteins were mainly localized in the nuclei and soma, and there was co-staining of the proteins. A qualitative increase of the immunolabeling for both factors was observed in RGCs treated with ET-1 or ET-3 (Fig 1).

### ET-1 induced upregulation of c-Jun and JNK and promoted AP-1 binding

**Fig 2:**
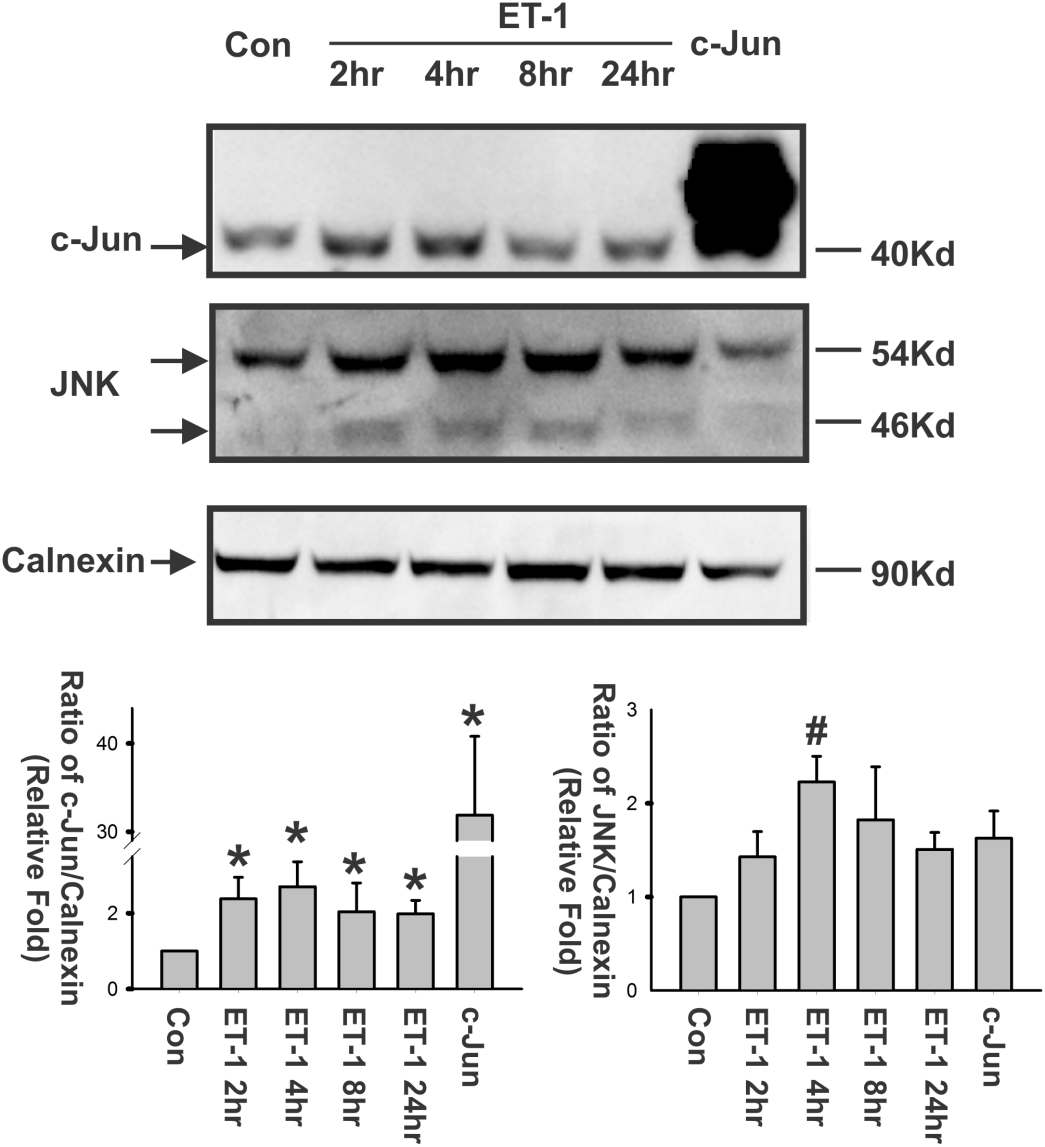
ET-1 induced upregulation of c-Jun and JNK. HNPE cells were treated with 100nM ET-1 at several time points, and cell lysates were subjected to detect c-Jun and JNK using western blot. Calnexin served as a loading control for total protein. ET-1 produced an increase of c-Jun and JNK expression from 2–24 hours post treatment. A set of representative data is shown from three repeats. Densitometry of western blot bands was analyzed using Bio-Rad Image Lab software. Bars represent mean and standard deviation (*: p< 0.05 and #: p< 0.01, one-way ANOVA, n = 3).

Since the yield of RGCs from rat retina is very limited and DNA transfection efficiency in primary RGCs is extremely low, we used HNPE cells for the following experiments similar to what was done in a previous publication [10]. The protein level of c-Jun was detected using western blot analysis. ET-1 induced an appreciable increase in c-Jun protein levels after ET-1 treatment in HNPE cells (Fig 2). The highest increase in c-Jun expression was observed at 2 and 4 hours post ET-1 treatment. c-Jun-N-terminal kinase (JNK), an upstream kinase of c-Jun, plays an important role to regulate the activity of c-Jun by phosphorylating c-Jun to phospho-c-Jun. In accordance with the c-Jun expression, increased JNK was detected post ET-1 treatment (Fig 2) and the significant upregulation of JNK was found at 4 hours post ET-1 treatment. This suggests that ET-1 enhances the protein levels of c-Jun as well as JNK.

**Fig 3:**
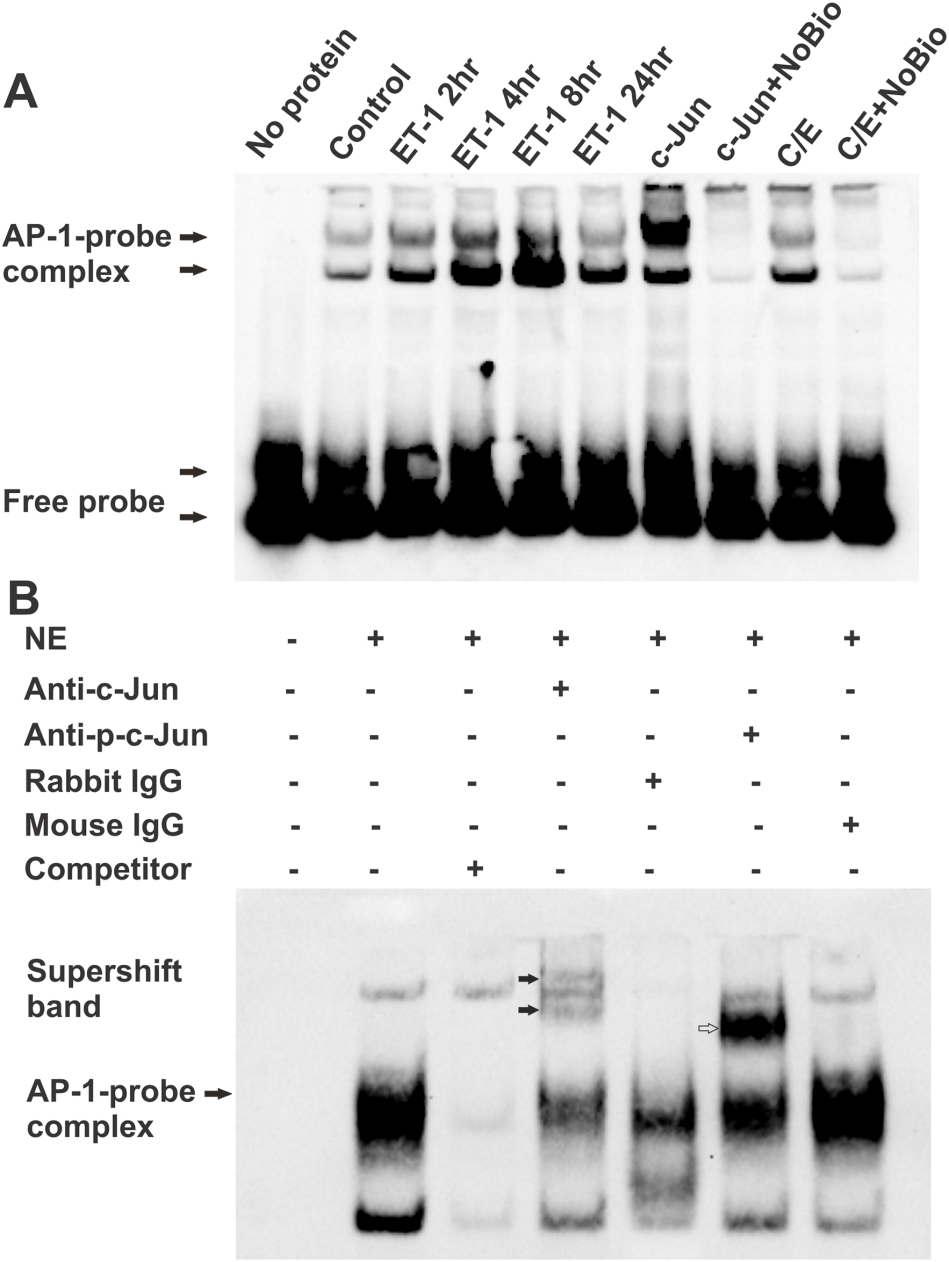
ET-1 treatment enhanced AP-1 binding. The nuclear fraction was isolated from HNPE cells following treatment with 100nM ET-1 at different time points. An EMSA assay was used to identify AP-1 DNA binding. (**A)** ET-1-induced c-Jun/AP-1 binding was tested using an EMSA assay. c-Jun overexpression served as a positive control for AP-1 binding, and the specificity of binding was confirmed by a binding reaction using non-biotin-labelled AP-1 oligonucleotides (NoBio) to compete the radio-labelled oligo. (C/E: C/EBPβ) A set of representative results was shown here from three repeats. (**B)** Supershift assay for EMSA was carried out by pre-incubation with either the phosphor-c-Jun antibody or the c-Jun antibody during the EMSA reaction system, and the specific binding of AP-1 complex was identified by the supershift (slow migration) of a complex of AP-1, labelled oligoes and antibody. Supershift is indicated with arrows in the figure (NE: nuclear protein extraction).

In addition to ET-1’s effects on the expression of c-Jun and JNK, c-Jun/AP-1 binding was tested using an EMSA assay in HNPE cells treated with ET-1 for 2–24 hours. It was found that the AP-1 DNA binding activity apparently started to increase at 2 hours post ET-1 treatment, reached the highest level at 8 hours, and persisted at high level to 24 hours (Fig 3A). The overexpression of c-Jun showed the enhanced binding, which was attenuated by competition using unlabeled AP-1 binding oligonucleotide (NoBio). Two AP-1 binding bands were identified in the EMSA assay, and the intensity of both bands was decreased when competitive unlabeled AP-1 binding oligonucleotide was applied, suggesting that both bands were specific. Other studies also reported similar findings [40–42]. The lower band was greatly increased by ET-1 treatment while the upper band was greatly increased by c-Jun overexpression. In order to confirm the specificity of AP-1 DNA binding, the reaction of AP-1 binding was pre-incubated with either the phospho-c-Jun antibody or the c-Jun antibody. In both cases the specific binding of antibody to AP-1 complex resulted the slower movement of whole complex in polyacrylamide gel and showed the resultant band shift (Fig 3B). As expected, the result was particularly striking with the phospho-c-Jun antibody, since c-Jun is phosphorylated before it is incorporated into the AP-1 complex.

### Overexpression of ET_A_ and ET_B_ receptor upregulated c-Jun

**Fig 4:**
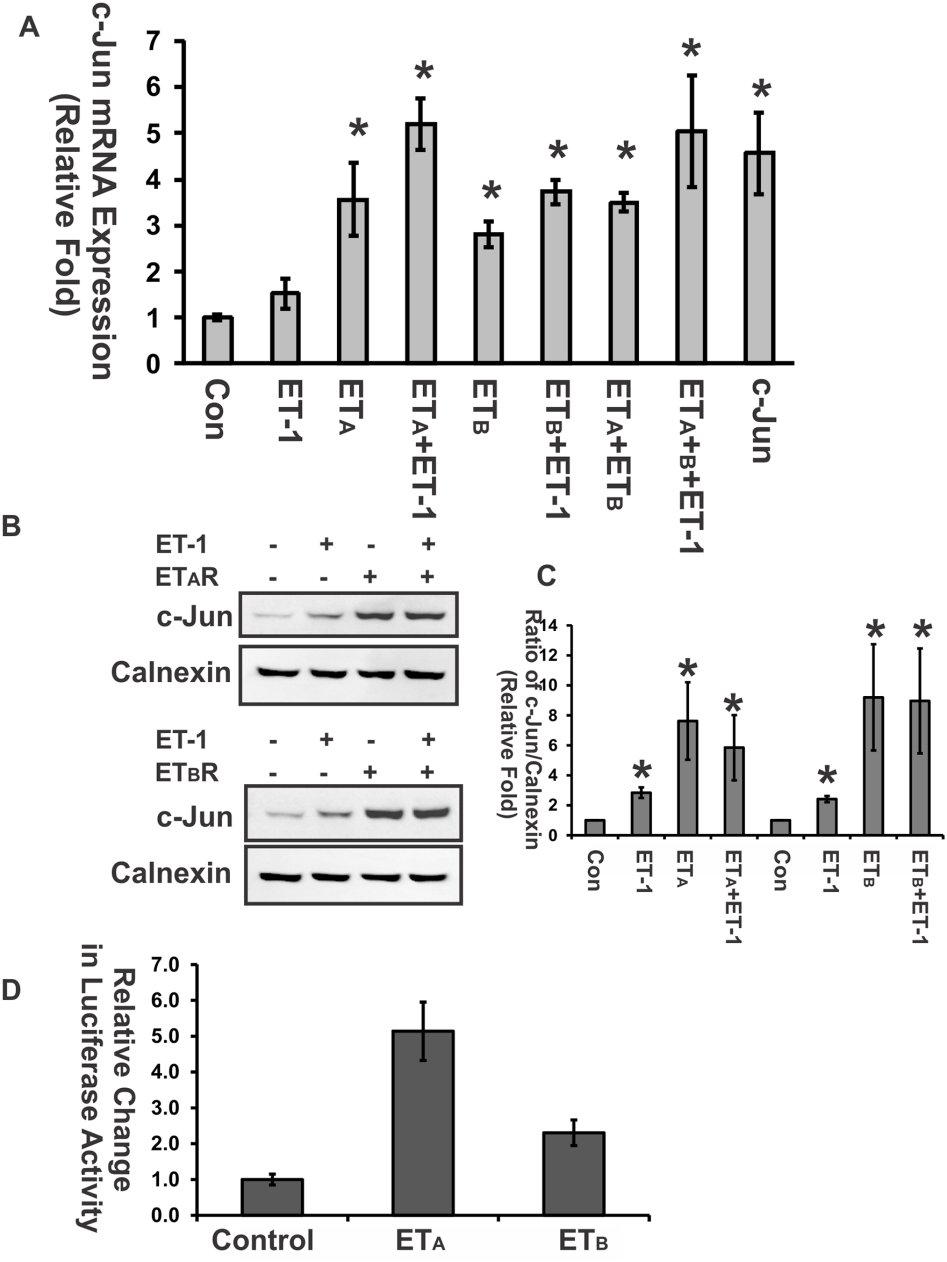
Overexpression of ET_A_ and ET_B_ receptor upregulated c-Jun expression. **(A)** SYBr-based-real-time PCR was used to detect c-Jun mRNA levels in HNPE cells treated with ET-1 with/without transient overexpression of the ET_A_ or ET_B_ receptor. Cyclophilin A served as an internal control, and the relative expression of genes was normalized to control (One way ANOVA, *: p<0.05, n = 3). (**B) & (C)** The protein levels of c-Jun were detected by western blot to confirm the results of real-time PCR. Calnexin served as a loading control for total protein. Densitometry of western blot bands was analyzed using Bio-Rad Image Lab software. Bars represent mean and standard deviation (*: p< 0.05, one-way ANOVA, n = 3). (**D)** c-Jun promoter region was cloned into pGL3 luciferase vector. Cell lysates from transiently co-overexpression of either ET_A_ or ET_B_ and pGL3-c-Jun vector in HNPE cells for 24 hours were used to detect luciferase activity. Luciferase reporter assay was carried out using Dual-Light System (Applied Biosystems, Bedford, MA) according to the manufacturer’s instruction. The luminescence was normalized by the protein amount of samples and triplicates were used in the assay. Luciferase activity was increased more than 5.1 fold in the group with ET_A_ receptor overexpression (* p<0.05, One way ANOVA, n = 3; Bars represent mean and standard deviation) and 2.3 fold in the group with ET_B_ overexpression compared to control which was transfected with luciferase construct without promoter.

Previously, we reported that overexpression of either c-Jun or C/EBPβ induces the upregulation of both ET_A_ and ET_B_ receptor [10]. Herein, we found that ET-1 treatment augments both c-Jun expression and AP-1 DNA binding ability; therefore, we investigated if c-Jun expression was induced by either direct activation or overexpression of the ET_A_ and ET_B_ receptor. The results from real-time PCR showed that c-Jun mRNA level was increased in the ET-1 treatment group, as well as in groups with transient overexpression of ET_A_ or ET_B_ receptors (One way ANOVA, p<0.05, n = 3) (Fig 4A). Correspondingly, c-Jun protein detection by western blot using samples with same treatments also confirmed this feed-forward regulation (Fig 4B & 4C). However, ET-1 treatment in HNPE cells with overexpression of ET receptors didn’t augment the further elevation of c-Jun protein expression. In addition, luciferase assay was carried out to detect c-Jun promoter activity which reflects the regulation of c-Jun gene. pGL3-c-Jun plasmid carrying the luciferase gene at the downstream of c-Jun promoter was used to detect ET receptors’ effects to enhance putative c-Jun promoter binding. The luciferase construct carrying c-Jun promoter was transiently co-transfected with either ET_A_ or ET_B_ expression vectors in HNPE cells for 24 hours. Luminescence intensity was detected immediately when cell lysate was prepared. The results showed that luciferase activity was increased more than 5.1 fold in HNPE with ET_A_ overexpression and 2.3 fold in HNPE with ET_B_ overexpression compared to that of control which was transfected with luciferase construct without promoter (p<0.05, One way ANOVA, n = 3) (Fig 4D). It suggests that overexpression of ET receptors (particularly, the ET_A_ receptor) induces some transcription factors that promoted c-Jun promoter binding.

### C/EBPβ binds to c-Jun promoter region

**Fig 5:**
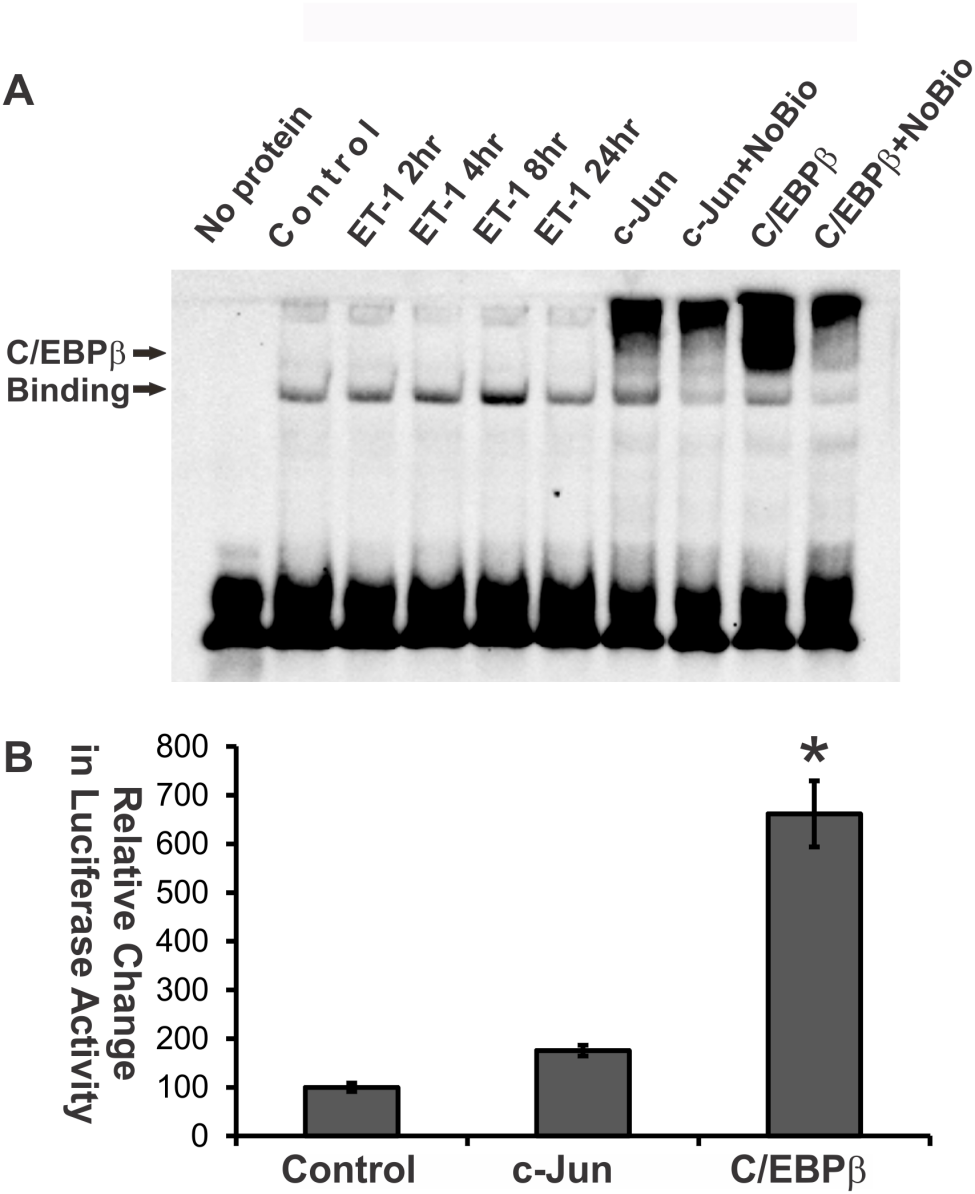
C/EBPβ binds to c-Jun promoter region and triggers c-Jun expression. **(A)** ET-1 treatment enhanced C/EBPβ binding. C/EBPβ binding was determined using EMSA assay. Overexpression of C/EBPβ served as a positive control and the specificity of binding was confirmed by a binding reaction using non-biotin-labelled C/EBPβ oligonucleotides (NoBio) to compete the radio-labelled oligos. (**B)** C/EBPβ binding to the c-Jun promoter region was determined by the luciferase-reporter assay using overexpression of C/EBPβ in HNPE cells. The relative luciferase activity was normalized by protein amount of the samples. (* p<0.05, One way ANOVA, n = 3; Bars represent mean and standard deviation).

**Fig 6:**
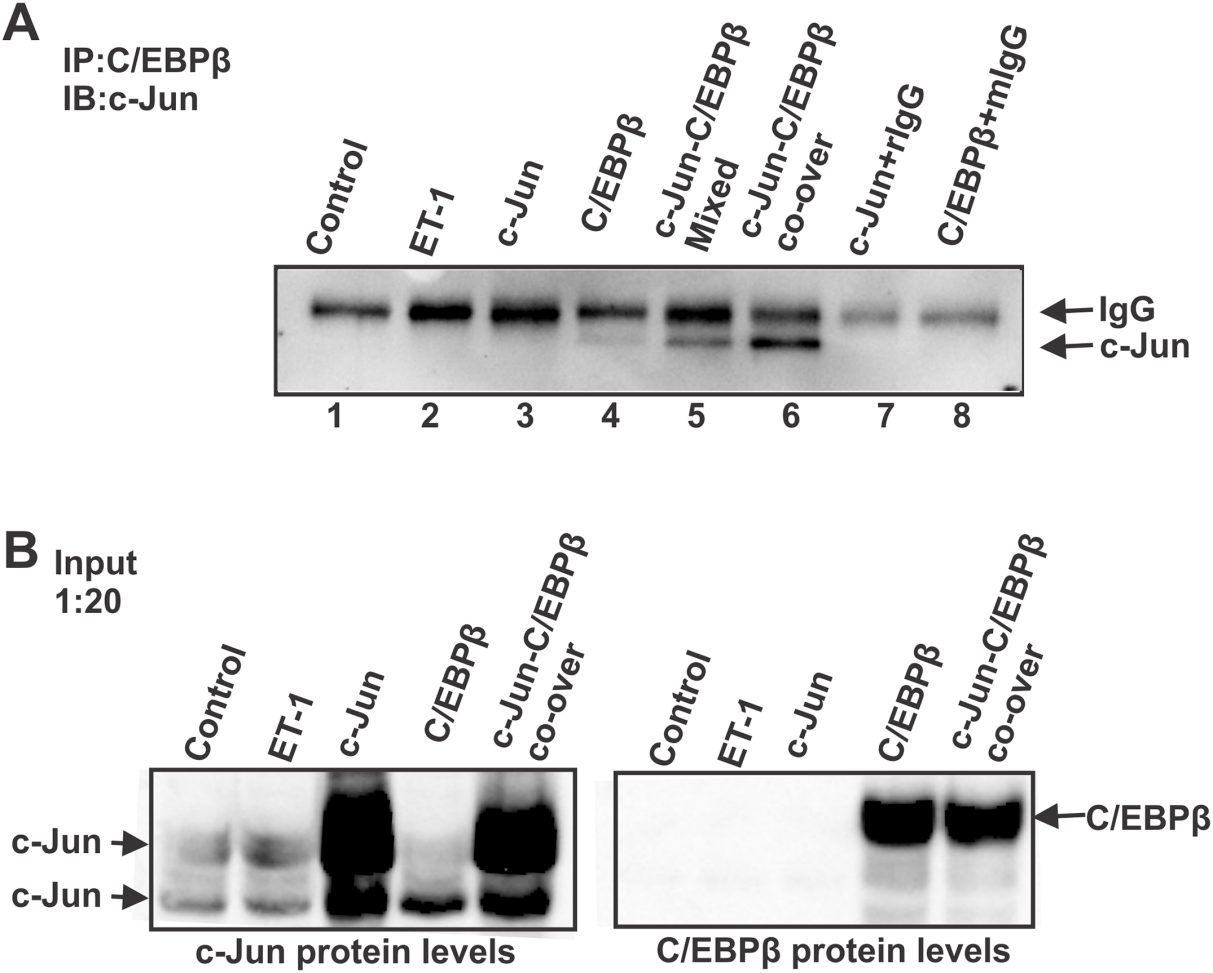
The interaction between c-Jun and C/EBPβ was confirmed using co-IP. **(A)** co-IP was carried out to confirm the physical interaction of c-Jun and C/EBPβ. The cell lysate was immunoprecipitated with mouse anti-C/EBPβ and subjected to western blot to detect c-Jun using a rabbit c-Jun antibody. Rabbit IgG or mouse IgG was incubated with samples from c-Jun or C/EBPβ overexpression to serve as a negative control. The representative result is shown from three repeats with a similar pattern. (**B)** One twentieth of the cell lysate amount that was used in co-IP was subjected to western blot to confirm the input proteins of c-Jun and/or C/EBPβ.

Since c-Jun and C/EBPβ were found to upregulate both ET_A_ and ET_B_ receptor expression in our previous study [10], we proposed that C/EBPβ could be one of factors regulating the expression of c-Jun. Therefore, we first tested if ET-1 induced an increase in C/EBPβ binding. The results from EMSA assays in HNPE cells showed that ET-1 treatment produced an increase in the DNA binding ability of C/EBPβ (Fig 5A), although endogenous C/EBPβ protein, even with ET-1 treatment, was not detectable using western blot (Fig 6B).

There are 19 C/EBPβ binding sites identified in c-Jun promoter region using the software Promo 3 (http://alggen.lsi.upc.es/cgi-bin/promo_v3/promo/promoinit.cgi?dirDB=TF_8.3). It was also found that an increase in AP-1 binding was detected using EMSA assays when C/EBPβ was overexpressed in HNPEs (Fig 3A), suggesting C/EBPβ might be an upstream regulator of c-Jun. Therefore, we investigated if C/EBPβ could regulate c-Jun expression through binding to the c-Jun promoter region. When C/EBPβ was co-expressed with the luciferase construct carrying c-Jun promoter region, the luciferase activity in co-expression group was increased 6.6 fold than that with the c-Jun promoter construct alone (p<0.05, One way ANOVA, n = 3) (Fig 5B). This finding suggests that C/EBPβ binds to the c-Jun promoter region and triggers the downstream gene upregulation represented by an increase in luciferase activity. Overexpression of c-Jun also produced an increase in the luciferase activity 1.7 fold; however, this result was not statistically significant (there are one binding site for AP-1, six for c-Jun, and one for c-Fos in c-Jun gene promoter based on analysis using Promo 3 software).

### The interaction between c-Jun and C/EBPβ was confirmed using co-immunoprecipitation

A previous report showed that both c-Jun and C/EBPβ were upregulated and co-localized at RGCs in response to an elevation of IOP[10]. ET-1 and ET-3 treatment also induced the upregulation of both factors in primary rat RGCs (Fig 1). In addition, our results suggested that C/EBPβ plays a regulatory role in c-Jun expression. In order to test the interaction of c-Jun and C/EBPβ, we carried out a co-immunoprecipitation (co-IP) assay to determine if there is a physical interaction of c-Jun and C/EBPβ. c-Jun and/or C/EBPβ was overexpressed in HNPE cells using transfection of their respective expression vectors. The cell lysate was incubated with mouse anti-C/EBPβ antibody and subjected to western blot to detect c-Jun using antibody raised from rabbit. The strongest signal obtained from co-IP was from the cell lysate prepared from co-expression of c-Jun and C/EBPβ (Fig 6A). An appreciable signal also came from the *ex vitro* mixture of the cell lysate with overexpression of c-Jun or C/EBPβ, respectively (Fig 6A). This suggests that there is an interaction and binding between c-Jun and C/EBPβ under physiological and *ex vitro* conditions. In addition, overexpression of C/EBPβ also generated a slight increase in the interaction between c-Jun and C/EBPβ. However, no co-IP pulldown signal was detected in groups with either ET-1 treatment or c-Jun overexpression due to the low abundance of endogenous C/EBPβ. Rabbit IgG or mouse IgG was incubated with samples from c-Jun or C/EBPβ overexpression to serve as a negative control. One twenty of total cell lysates that were used in co-IP was subjected to western blot to confirm the protein expression and the input amount used in co-IP (Fig 6B). c-Jun protein was detected in all conditions, and appreciably increased in ET-1 treatment and in the C/EBPβ overexpression group. However, C/EBPβ was detectable only in the C/EBPβ overexpression group and endogenous protein was not detectable due to low abundance (Fig 6B).

### ET-1 induced the apoptosis of HNPE

**Fig 7:**
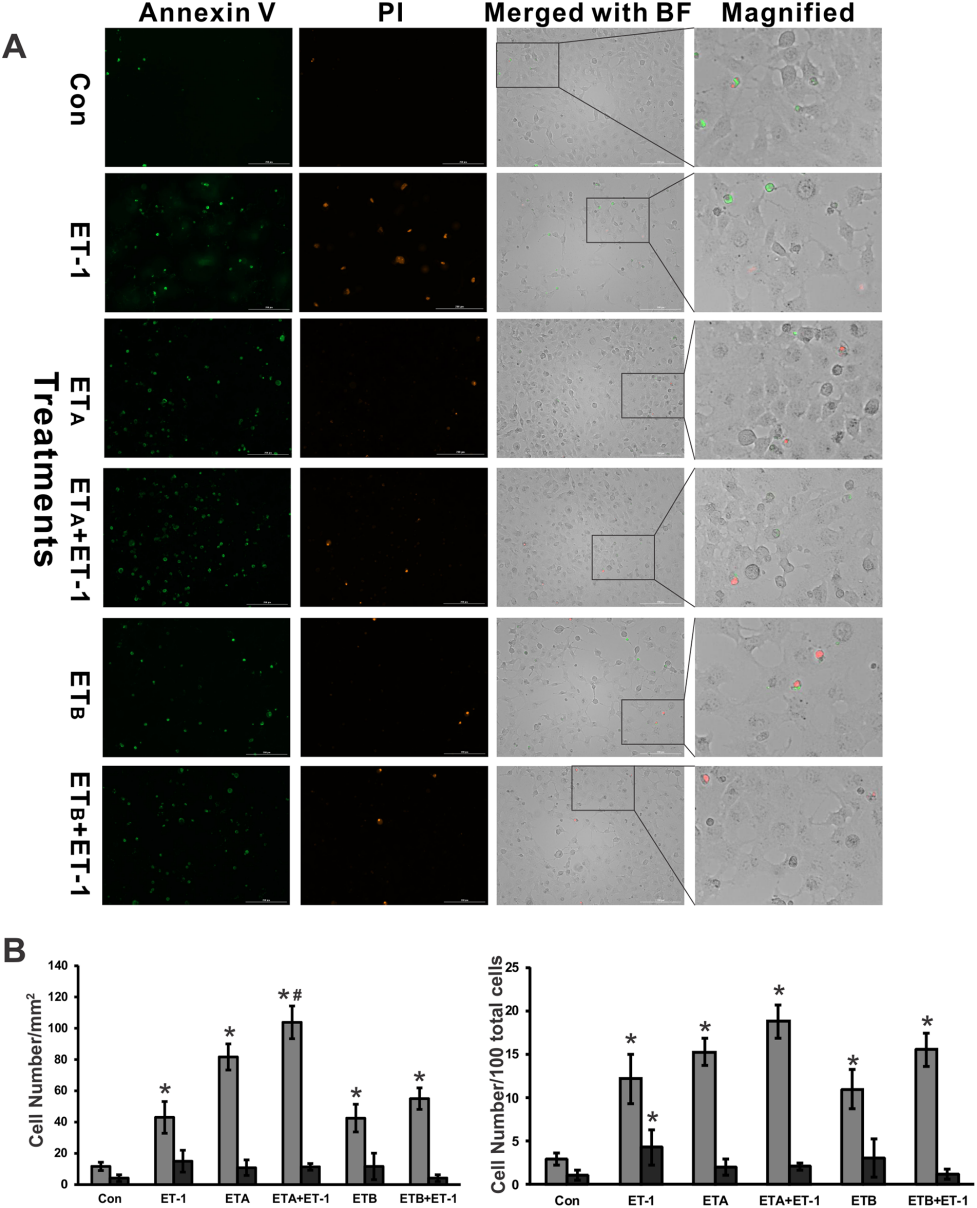
ET-1 induced the apoptosis of HNPE cells. HNPE cells treated with 100nM ET-1 for 24 hours induced greater apoptosis compared to control. (**A)** ET-1-induced apoptosis or necrosis in HNPE cells was determined using Annexin V/PI staining (Biotool, Inc. Houston, TX). Images were captured using the Cytation 5 (BioTek, Inc.). Green staining represents apoptosis from Annexin binding and red staining is indicative of necrosis from PI binding. A partial region of merged images was shown at higher magnification. (**B)** The apoptotic, necrotic cells, and total cells were counted and the results were shown as cell number/mm2 and cell number/100 total cell number. The significance was analyzed using One-way ANOVA. Grey bar represents apoptotic cell counting and black bar represents necrotic cell counting. (*, # represents p<0.05, n = 4. *: versus control; #: receptor overexpression + ET-1 treatment versus receptor overexpression).

ETs exert profound biological functions in a variety of cells. It induces apoptosis in RGCs[8], and in contrast promotes cell proliferation in astrocytes[43, 44]. Therefore, we determined if ET-1 induces apoptosis or necrosis in HNPE cells using Annexin V/Propidium Iodide (PI) staining. ET-1 treatment for 24 hours induced apoptosis compared to that seen in the control (Fig 7; * p<0.05 compared to control, One-way ANOVA, n = 4). Overexpression of ET_A_ or ET_B_ receptor also promoted cell apoptosis (Fig 7; * p<0.05 compared to control, One-way ANOVA, n = 4). A combination treatment of ET-1 and ET_A_ receptor overexpression induced even a greater number of apoptotic cells (Fig 7; # p<0.05, ETA+ET-1 vs ET-1, One-way ANOVA, n = 4). In addition, there was no significant difference in necrotic cell number/mm^2^ (PI staining) among other groups (Fig 7B); however, ET-1 induced a slight increase of necrosis (p = 0.044) compared to control when dead cell ratio (necrotic cell number/100 total cells) was taken into consideration.

## Discussion

ET_A_ and ET_B_ receptors belong to the rhodopsin family of GPCRs. ET-mediated signaling pathways occur through ET_A_ and ET_B_ receptors coupling with Gα_i_, Gα_s_, Gαq and Gα12/13 [45]. It has been suggested that several transcription factors, such Elk-1, c-Fos, c-Jun, ATF-4, and AP-1 [28, 33, 34], play a gene regulatory role in several signaling pathways. In the current study, we investigated the relationship between ET receptor and c-Jun.

Endothelins (ETs) and their endothelin B (ET_B_) receptors are emerging as important players in neurodegeneration in glaucoma. For example, mRNA and protein levels of ET_B_ receptors were increased in ocular tissues subjected to glaucomatous insults [8, 10, 46–49]. Previous studies also showed that upregulation of c-Jun and C/EBPβ occur in the ganglion cell layer in the rat retina in response to elevated IOP[10] and ET-1 treatment promotes c-Jun and phosphorylated c-Jun in RGCs[35]. Blocking the JNK-c-Jun pathway also generates a neuroprotective effect. Prevention of RGC death from optic nerve crush was reported in JNK2/3 deficient mice[26], and IOP-mediated RGC death and axon loss was also alleviated in rats by administration of the JNK inhibitor SP600125 [25]. These findings suggest that c-Jun plays a crucial role in neurodegeneration of RGCs. In addition, the transcription factors, c-Jun and C/EBPβ, were found to bind to the upstream promoter region of the ET_B_ receptor and produced the upregulation of these receptor expression. Overexpression of either of the two factors induced an increase in mRNA level of ET_A_ and ET_B_ receptors[10]. Increased immunohistochemical staining of c-Jun and C/EBPβ in ganglion cells at the retina in response to elevated IOP were also associated with an increase in immunostaining and mRNA levels of the ET_B_ receptor. Overexpression of both factors induces an increase in mRNA levels of ET_A_ receptors[10]. In this current study, the relative contribution of the two ET receptors on the regulation of c-Jun was addressed. Although several studies and reviews have shown that ETs induce the upregulation of c-Jun [50–53], ET’s actions within the eye are still not fully understood. Herein, we demonstrated that ET-1 treatment induced upregulation of c-Jun mRNA and protein levels in both primary rat ganglion cells [35] and HNPE cells (Figs 1, 2 and 3). Functional AP-1 binding ability was also confirmed using EMSA assays in the current study. Furthermore, overexpression of ET receptors produced an appreciable increase of c-Jun in both mRNA and protein levels. Therefore, our results suggest that there is a positive feed-forward loop in the regulation between ET receptors and c-Jun in HNPE cells. ET-1 binds to its receptors and the resultant activation of ET receptors upregulates c-Jun through a variety of signaling pathways [28, 33, 34, 50, 51]. c-Jun combines with c-Fos, c-Jun, ATF-3 to form AP-1, which binds to the promoter region of ET receptors to upregulate receptor expression.

Another important observation from the current study was that we identified the direct interaction between c-Jun and C/EBPβ, and also demonstrated that C/EBPβ was an upstream regulator of c-Jun. It is noted that c-Jun/AP-1 and C/EBPβ contain the conservative bZip domains, raising the possibility that both proteins interact with each other as commonly seen in bZip domain-containing proteins [54–56]. A close relationship was demonstrated in studies for the structure of the basic leucine zipper (bZIP) region [15, 54, 56]. Although it’s also reported that a member of C/EBP was associated with c-Fos and c-Jun [15], there is no clear evidence to prove the direct interaction between c-Jun and C/EBPβ. Other studies showed that C/EBPβ co-operated with c-Jun and bound to the promoter region to trigger and regulate gene expression [17, 57, 58]. It has been reported that c-Jun functioned as a coactivator with a complex of the ETS transcription factor PU.1 and C/EBPβ, and the physical interaction and binding of c-Jun was tested by a GST affinity pulldown assay[59], however, no direct interaction between c-Jun and C/EBPβ was determined. Our previous study and other reports showed that c-Jun/AP-1 and C/EBPβ played important roles in regulating ET_A_ receptor and ET_B_ receptor expression in HNPE and vascular smooth muscle cells [10, 60, 61]. In this current study, we used a co-IP assay to identity protein-protein interactions from two different samples: *ex vitro* mixed cell lysates containing overexpressed c-Jun and C/EBPβ respectively and cell lysates with co-expression of both factors. Our results indicate that c-Jun and C/EBPβ are co-localized in the cells and that they have direct interactions with each other. In addition, they appear to regulate each other’s expression. Wang et. al. showed that the knockdown of JNK2 activity didn’t affect the protein levels of C/EBP-β [62]. However, a dominant-negative of JNK1 and attenuation of c-Jun inhibited C2-ceramide-induced-expression of C/EBPβ [63]. In our current study, we focused on the mechanism by which c-Jun was regulated. Interestingly, we found that there are 19 DNA binding sites of C/EBPβ identified in a 1043-bp upstream region of c-Jun promoter using Promo 3 software. Overexpression of C/EBPβ promoted its binding to the promoter region of c-Jun, leading to enhanced transcription of c-Jun. Furthermore, overexpression of ET receptors also enhanced luciferase activity following promoter binding. It is not clear if the factors other than C/EBPβ are also involved. Moreover, C/EBPβ can also influence gene regulation in concert with SF-1 and ATF-4 [64]. It is reported that C/EBPβ directly interacts with cyclic AMP-induced forkhead transcription factor (FKHR) to form a complex to regulate gene expression in the differentiation of human endometrial stromal cells[65]. Thus, these transcription factors appear to provide a wide range of gene regulation.

Initially discovered as a potent vasoactive peptide, ET-1 has vasoconstriction or vasodilation effects that are dependent on the expression of ET_A_ and ET_B_ receptors in the blood vessel involved. Growing evidence has shown that ET-1 is involved in numerous cellular effects including development, cell differentiation, cell proliferation, tumor metastasis, angiogenesis, electrolyte balance, matrix formation, mitogenesis, acute or neuropathic pain, apoptosis and anti-apoptosis [33, 66–78]. The diverse actions of ET-1 can be illustrated in terms of the expression of ET-1, the existence of several receptor subtypes, the differential expression of receptors in cells and tissues, and the activation of a variety of different signaling pathways. One of main sources of ET-1 is from endothelial cells, however ET-1 is also produced by many other types of cells, including epithelial cells, macrophages, fibroblast, cardiomyocytes, brain neurons and ocular cells [72, 74, 75, 79]. In eyes, aqueous humor is produced and secreted by the ciliary body, and ET-1 is synthesized and released from HNPE cells [80, 81]. In our previous studies, ET-1 induced cell proliferation in a human astrocytoma cell line (U373MG)[43], primary human optic nerve head astrocytes[44], and triggered apoptosis of primary rat RGCs[8]. In the current study, we investigated the role of ET-1 on HNPE cells. We found that ET-1 treatment induced apoptosis of HNPE cells. Overexpression of ET_A_ and ET_B_ receptors also induced greater apoptotic changes, however, no noticeable difference of PI-labelled necrosis was identified. Annexin V staining represents the early stage of apoptosis, therefore, apoptosis of HNPE cells caused by ET-1 treatment might be an immediate response to attenuate the release of ET-1 to prevent further cell damage. Our findings suggest that c-Jun plays a feed-forward regulatory role in expression of ET receptors in HNPE cells. RGCs are ET-1-targeted cells with expression of both ET_A_[82] and ET_B_ receptors [8], and ET-1 treatment increased protein and mRNA levels of c-Jun and ET receptors in the cells [35], it is possible that this feed-forward role of c-Jun would be a similar process in RGCs; however, further studies are needed to confirm this.

**Fig 8:**
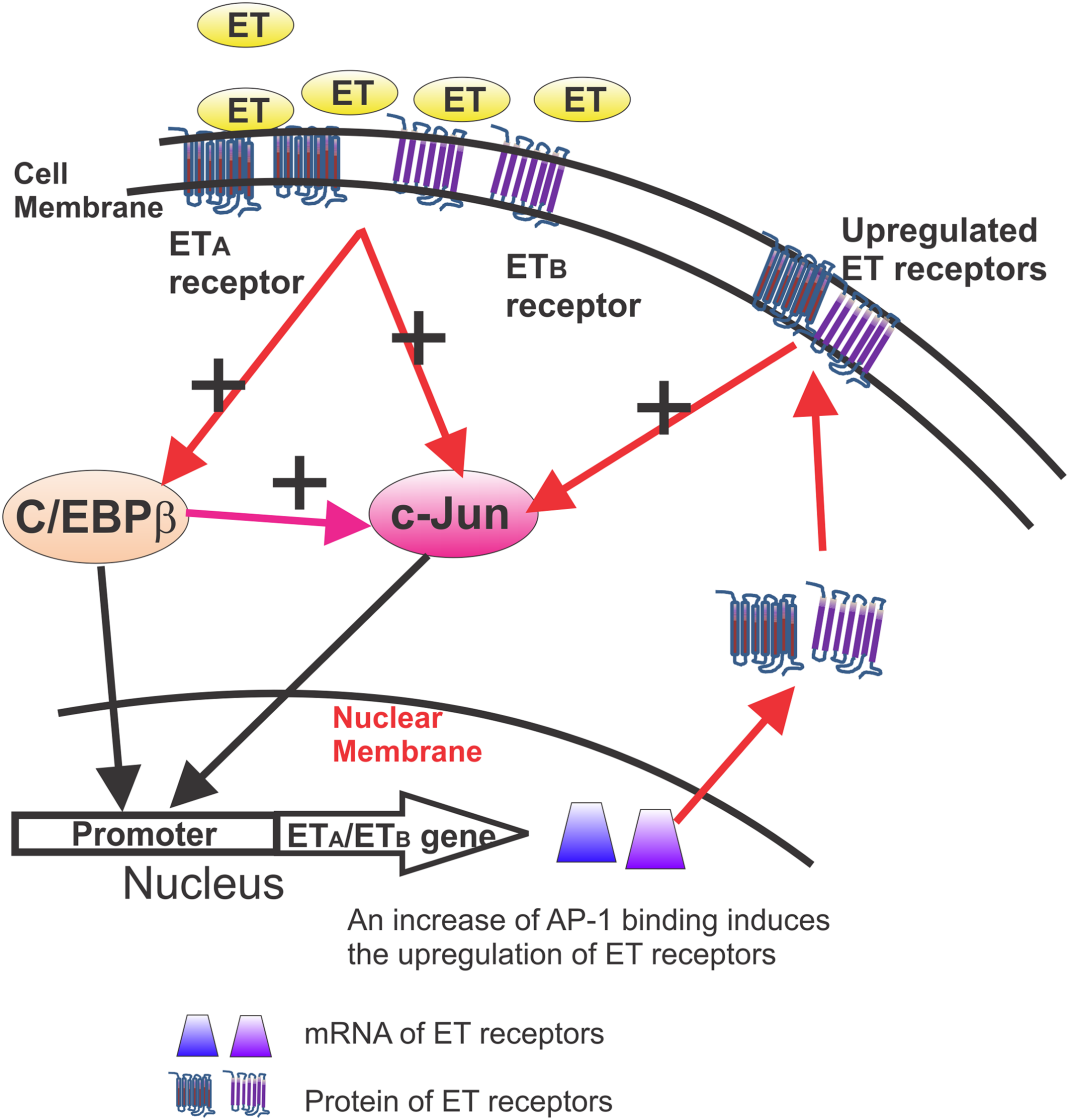
A diagram depicting a feed-forward regulatory loop between ET receptor and c-Jun expression. ET-1 binds to ET_A_ and ET_B_ receptors and activates them, leading to the upregulation of c-Jun and C/EBPβ expression and an increase in DNA binding ability of both transcription factors. Moreover, c-Jun and C/EBPβ were found to promote the expression of ET_A_ and ET_B_ receptors. The direct interaction between c-Jun and C/EBPβ was also firstly reported in this study. C/EBPβ plays a regulatory role in expression of c-Jun. In addition, overexpression of ET_A_ and ET_B_ receptors induces the upregulation of c-Jun as well. Taken together, our results suggest that ET-1 triggers the upregulation of c-Jun through both receptors, and conversely c-Jun also has a feed-forward role in elevating endothelin receptor expression.

In summary, ET-1 induced an increase in the upregulation of c-Jun and the DNA binding of c-Jun and C/EBPβ. The direct interaction between c-Jun and C/EBPβ was also reported for the first time in this study. C/EBPβ was also found to play a regulatory role in the expression of c-Jun, while c-Jun was previously shown to regulate ET_A_ and ET_B_ receptors. Taken together, our results suggest that ET-1 triggers the upregulation of c-Jun through both ET receptors, and that conversely c-Jun also has a feed-forward role in elevating endothelin receptor expression (Fig 8). Such activities can lead to neurodegeneration, and a better understanding of the gene regulation and signaling of endothelin receptors could aid in the development of neuroprotective agents.

## Supporting information

S1 File Supplimental data for western blot of JNK. (PDF)
