# Supplementary material for: A feed-forward regulation of endothelin receptors by c-Jun in human non-pigmented ciliary epithelial cells and retinal ganglion cells
Source: PLoS One. 2017 Sep 22;12(9):e0185390. doi: 10.1371/journal.pone.0185390 (PMC5609771; doi:10.1371/journal.pone.0185390)
Supplement: S1 File — (PDF) [file pone.0185390.s001.pdf]

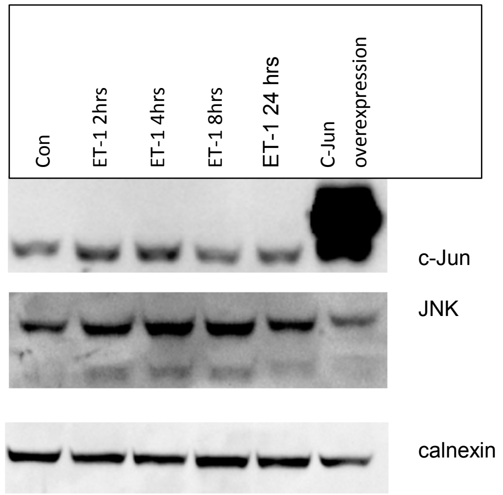

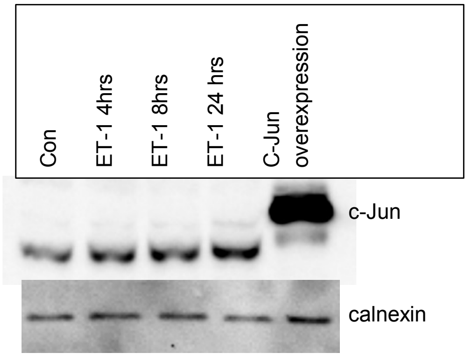

*JNK was not detected in this set

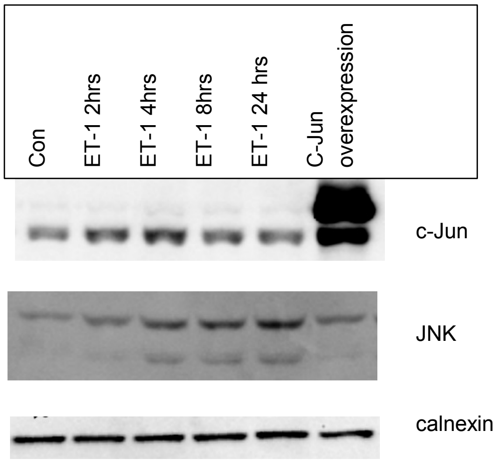

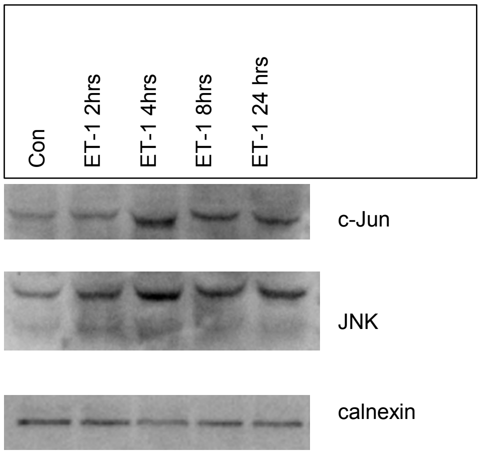

c-Jun, JNK and loading control (calnexin) were detected in three/four biological replicates by western blot. The summary of data was shown in this supplemental file.
